# A retrospective evaluation on the effects of a *Bacillus*-based probiotic supplementation on milk yield, milk composition, health status, and reproductive performance of dairy cows in Argentina

**DOI:** 10.1093/tas/txag144

**Published:** 2026-09-21

**Authors:** Elias Giugge, Fernando M Clemente, Melina Briosso, Oscar C M Queiroz, Jens N Joergensen, Bruno I Cappellozza

**Affiliations:** Grupo Chiavassa, Santa Fé S2453, Argentina; Novonesis, Buenos Aires C1107, Argentina; Novonesis, Buenos Aires C1107, Argentina; Novonesis, Hørsholm 2970, Denmark; Novonesis, Hørsholm 2970, Denmark; Novonesis, Hørsholm 2970, Denmark

**Keywords:** *Bacillus* spp, dairy cows, health, milk production

## Abstract

The objective of this retrospective before-and-after trial was to evaluate the effects of a *Bacillus*-based direct-fed microbial *(DFM)* on milk yield, composition, reproductive performance, and health status of dairy cows in a single commercial dairy farm in Argentina. The dataset used in the current study comprised January 2019 until September 2025, including roughly 1,300 to 1,600 lactating Holstein cows across the study period, split into 10 pens. The data between January 2019 to September 2023 was considered as the period pre-DFM feeding (57 mo; *PRE*), whereas the period of DFM supplementation (*B. licheniformis* 809 and *B. subtilis* 810) considered in the analysis ranged from October 2023 to September 2025 (24 mo; *POST*). During this period, the *Bacillus*-based DFM was fed at 3 g/cow per day. Milk yield was evaluated daily and individually for all lactating cows, while milk composition was evaluated monthly for the whole-farm. Based on these results, 4% FCM and ECM yield were determined. Moreover, reproductive and health data were also collected. All data were analyzed with the GLIMMIX and GLM procedure of SAS, considering pen as the experimental unit. No treatment × season interactions were observed for any of the productive and health variables (*P* ≥ 0.08). Treatment group and season were associated with differences in milk yield (*P* ≤ 0.02). Milk yield was 1.0 kg/cow per day greater (*P* = 0.02) in POST cows, while 4% FCM and ECM were 3.6 and 4.1 kg/day greater (*P* ≤ 0.01), respectively, following the DFM supplementation. Cows in the winter and spring produced more milk (*P* < 0.01) than in the fall and summer. For milk composition (kg and %), positive changes were observed and associated with the DFM supplementation period (*P* ≤ 0.07). The occurrence of adverse heath events and mortality rate were observed to be reduced (*P* < 0.001) from 32.8 and 1.6% to 23.7 and 0.7% for PRE and POST periods, respectively. Among the individual adverse health outcomes, mastitis, ketosis, metritis, displaced abomasum, and pneumonia occurrence were significantly reduced in POST vs. PRE (*P* ≤ 0.02). Treatment group × season interactions were observed for both conception and abortion rates (*P* ≤ 0.03); however, the interactions were noted mainly because of the season effect, given that within the same season, no treatment differences were observed (*P* ≥ 0.14). No treatment effects were observed either on conception or abortion rate (*P* ≥ 0.31). In summary, in this retrospective before-and-after study, *B. licheniformis* 809 and *B. subtilis* 810 supplementation was associated with an improved milk production, composition, and the health of the herd, while maintaining the reproductive performance of the commercial farm in Argentina.

## Teaser

This retrospective before-and-after study reported the potential benefits of *Bacillus*-based probiotics on milk production, milk solids composition, and health status of lactating dairy cows over 24 months.

## Introduction

In dairy cattle, direct-fed microbials (*DFM*) based on live bacteria have been extensively evaluated as a manner to support rumen health and gut health, nutrient digestion, milk production, and overall efficiency of the herd (El Jeni et al. 2024). As an example, the supplementation of rumen-native live microorganisms has benefited milk yield and feed efficiency of post-partum dairy cows (Valldecabres et al. 2022; Nehme Marinho et al. 2024). More recently, the utilization of *Bacillus* spp. has been increasing in livestock segments, given their spore-forming trait (Bernardeau et al. 2017) and broad mode of action that supports both the health and performance of ruminants (Luise et al. 2022; Cappellozza et al. 2025). In fact, *B. subtilis* supplementation has increased milk and solids yield in dairy cows during late-lactation, but not in early-lactating cows (Souza et al. 2017). In dairy cows, a combination of *B. licheniformis* and *B. subtilis* supplementation improved milk yield (Cappellozza et al. 2024; Bach et al. 2026; Nicola et al. 2026), feed efficiency (Cappellozza et al. 2024; Terré et al. 2024; Bach et al. 2026), and milk composition (Cappellozza et al. 2024; Arthur et al. 2025; Bach et al. 2026) in early-, mid-, and late-lactating cows.

The validation of research-proven, scientific-based solutions in the field (i.e. dairy farm), over an extended period of time, is paramount for further adoption of technologies by the livestock segment. The day-to-day routine of a dairy farm, associated with all the environmental changes, genetics, management, and nutrition may pose some additional challenges that really test the real results of a specific technology. Hence, one alternative to create a more comprehensive and reliable dataset that allows researchers to dilute and minimize the effects of a specific season or year on the utilization of a technology in the farm would be to gather data from several years in the same farm. To our knowledge, few long-term on-farm assessments have been published evaluating technologies applied to the dairy farms. Based on this rationale, the current retrospective before-and-after study gathered approximately 7 years of data regarding milk yield, milk composition, reproductive performance, and health for further statistical analysis and assessment of the yearly evolution of a dairy farm. Moreover, the supplementation of a mixture of *Bacillus* spp. for extended periods of time (>15 wks) is limited and warrants further investigation. Hence, we hypothesized that feeding *B. licheniformis* 809 and *B. subtilis* 810 would be associated with improved performance, health status, and reproductive function of dairy cows in a commercial farm in Argentina. Therefore, our objective was to assess the effects of *Bacillus* spp. supplementation on milk yield, composition, reproductive function, and health status of a dairy cow herd under a before-and-after retrospective study.

## Materials and methods

### Animals, housing, and management description

The commercial dairy farm (Grupo Chiavassa) is located in Carlos Pellegrini, Santa Fé, Argentina (latitude 32°3’S, longitude 61°48 W, and altitude 101 m). The dataset used in the current study comprised January 2019 until September 2025, including a total of 1,468, 1,377, 1,598, 1,480, 1,572, 1,398, and 1,465 primiparous and multiparous lactating Holstein cows per year, split into 10 pens, respectively. The data between January 2019 to September 2023 was considered as the period pre-DFM feeding (57 months; *PRE*), whereas the period of DFM supplementation (Bovacillus; Novonesis, Puerto Madero, Buenos Aires, Argentina) considered in the analysis ranged from October 2023 to September 2025 (24 months; *POST*). The 57 mo for data evaluation was to account for any potential difference between years and also to provide a better confidence in the retrospective analysis performed herein. During POST, the *Bacillus*-based DFM was fed at 3 g/cow per day (9.6 × 10^9^ CFU/head per day; 3.2 × 10^9^ CFU/g) via the commercial mineral-vitamin premix (Cooperativa Guillermo Lehmann, Pilar, Santa Fé, Argentina). The aforementioned dose of DFM is the standard recommendation of the manufacturer, which has also been evaluated in previous scientific published studies (Cappellozza et al. 2024; Arthur et al. 2025; Bach et al. 2026). During the period of evaluation, all cows were housed in free-stall barns with an evaporative cooling system consisting of fans and sprinklers in the feed bunk, while the proportion of multiparous and primiparous cows was 73% and 27% and 74% and 26% for PRE and POST, respectively. Over the years, the standard TMR contained whole-plant corn silage, grass silage, wheat silage (2023 to 2025), and a concentrate mix. The concentrate mix was composed of ground corn, soybean meal, whole cottonseed, high-moisture corn grain, rumen-protected soybean meal, limestone, acid buffer, and a mineral-vitamin premix. The concentrate mix contained at least (DM basis) 62% of corn grain and high-moisture corn, 32% of soybean meal, whole cottonseed, and rumen-protected soybean meal, and 6% limestone, acid buffer, and the mineral-vitamin premix. The TMR was formulated to meet the requirements of the cows in each pen (NASEM 2021) and an overview of the diets in each year is described in Table 1.

**Table 1:** Overview of the composition and nutritional profile of the diets offered to the dairy cows from 2019 to 2025.

Year	kg dry matter (DM)					Nutritional profile								
	Wheat silage	Whole-plant corn silage	Grass silage	Concentrate mix^a^	Total^b^	DM, %	CP, % DM	Starch, % DM	NDF, % DM	ADF, % DM	Fat, % DM	ME, Mcal/kg	NE_m_, Mcal/kg	NE_L_, Mcal/kg
**2019**	—	8.93	6.00	11.1	26.0	45.5	18.0	25.4	32.6	20.4	5.7	2.77	1.81	1.69
**2020**	—	9.44	6.02	13.7	29.2	45.0	15.9	24.9	33.2	19.8	4.8	2.72	1.79	1.68
**2021**	—	10.9	7.33	11.0	29.2	45.0	16.4	26.6	33.3	20.5	3.9	2.75	1.79	1.68
**2022**	—	11.0	7.05	11.1	29.2	46.0	16.4	26.9	33.0	20.2	3.9	2.75	1.79	1.68
**2023^c^**	0.75	7.25	8.58	12.6	29.2	45.0	16.9	26.4	32.4	20.1	4.0	2.75	1.79	1.68
**2023^d^**	3.40	7.10	5.00	13.8	29.3	48.0	16.7	26.3	33.8	20.7	4.0	2.74	1.79	1.68
**2024**	2.91	6.47	5.25	14.4	29.0	46.0	17.0	26.2	31.8	19.8	3.8	2.76	1.80	1.69
**2025**	2.80	6.95	5.98	13.5	29.2	45.0	16.8	26.0	32.6	20.1	4.1	2.75	1.79	1.68

a Concentrate mix contained corn grain, soybean meal, whole cottonseed, high-moisture corn grain, rumen-protected soybean meal, calcium carbonate, and a mineral-vitamin premix. In the nutritional profile analysis, the composition from each year was evaluated separately and results are presented here.

b Total DM offered.

c Data from January to September 2023.

d Data from October, November, and December 2023.

### Sampling

Throughout the period of evaluation, cows were milked three times per day, milk yield was evaluated daily and individually for all cows (De Laval SA, Santa Fe, Argentina), while the variation in milk yield (kg/day) was determined as the difference of milk yield between consecutive days. Milk composition (fat, protein, SNF, total solids, and SCC) was evaluated monthly for the whole-farm as a bulk tank sample, resulting in one sample per month. Based on these results, 4% FCM was determined using the equations proposed by Gaines and Davidson (1923), while ECM was estimated with Tyrell and Reid (1965). Temperature and humidity data were also collected daily throughout the years for determination of the temperature-humidity index (*THI*; Mader et al. 2006) and the threshold of THI = 68 was used to classify cows with heat stress (Ouellet et al. 2021).

Over the years, health and reproductive data were collected and recorded from 2021 to 2025 according to veterinary records. Health data were stratified according to the adverse health events, including metritis (based on vaginal discharge; Garzon et al. 2022), mastitis (based on SCC; Fernandes et al. 2021), retained placenta (failure to expel the fetal membranes within 24 hours after calving; Haimerl and Heuwieser 2014), lameness (visual locomotion score; Schlageter-Tello et al. 2014), ketosis (beta-hydroxybutyrate determination post-calving; McArt et al. 2012), hypocalcemia (based on blood calcium concentration around calving; Oetzel 2004; Oetzel and Miller 2012), pneumonia (McCarthy et al. 2021), and displaced abomasum (Le Blanc et al. 2005). All the diseases were recorded and diagnosed according to the farm standard operational procedures, while the reproductive data analyzed included conception [number of cows pregnant per cows exposed to the timed-artificial insemination (*TAI*) procedure] and abortion (number of cows calving per cows pregnant at the pregnancy diagnosis procedure at 30 days post-TAI) rates.

### Statistical analysis

All data were analyzed with the SAS software (version 9.4; SAS Inc., Cary, NC). The data were summarized, within year, into seasons (autumn, winter, spring, and summer) and according to the THI, classified as heat stress (*HS*; 0 = no HS and 1 = HS). Pen was used as the experimental unit, the exception being the data for milk composition, which used the month as the experimental unit. The continuous variables were analyzed with the GLIMMIX procedure of SAS, considering pen, year, HS, and month as the random factors, treatment groups (PRE and POST), season and the resultant interaction as fixed factors. Groups of pens that were entering the farm between the third trimester of 2023 and 2025 were not used in the current evaluation, given that a representative group prior to the DFM supplementation would not be available. The containment method to determine the degrees of freedom for the fixed effects was used. Somatic cell count was log-transformed for the statistical analysis and then back-transformed for reporting as × 1000 cells/mL. All results are reported as least-square means and adjusted for Bonferroni. The Bonferroni adjustment (ADJUST = BON) was applied to control the family-wise Type I error rate for the multiple pairwise comparisons requested by the PDIFF option. The occurrence of adverse health events, mortality rate, conception rate, and abortion rate were analyzed using the GLM procedure of SAS for continuous proportion outcomes. Therefore, no non-normal distribution or link function was specified; the analyses assumed normally distributed residuals and used the identity link inherent to the general linear model. Pairwise comparisons among least-squares means were adjusted using the Bonferroni method. For all the data, significances were set at *P* ≤ 0.05 and tendencies were denoted if 0.05 < *P* ≤ 0.10.

## Results

Table 1 presents the overall composition and nutritional profile of the diets over the retrospective analysis. No drastic changes were observed in the nutritional profile of the diets, while variations in the composition of the diet are expected due to the fluctuations in the price and availability of the raw materials. Table 2 presents the overall descriptive analysis of all the productive variables analyzed herein at the commercial dairy farm from 2019 to 2025, while descriptive health and reproductive data are reported from 2021 to 2025. Moreover, during the PRE and POST periods, 30.0 (17/57) and 37.5% (9/24) of the months had an average THI > 68, respectively, suggesting the occurrence of HS.

**Table 2:** Descriptive analysis (mean ± standard deviation) of the variables analyzed in the current before-and-after retrospective study, from 2019 to 2025.

Item	2019	2020	2021	2022	2023^a^	2023^b^	2024	2025
**Milk yield, kg/day**	33.5 ± 5.45	36.1 ± 7.71	35.7 ± 6.68	35.5 ± 6.58	36.4 ± 6.54	38.1 ± 6.54	36.1 ± 6.62	35.2 ± 6.80
**Milk solids, kg/day**								
** Fat**	1.49 ± 0.116	1.64 ± 0.156	1.68 ± 0.197	1.86 ± 0.135	1.87 ± 0.195	1.96 ± 0.111	1.89 ± 0.134	1.94 ± 0.185
** Protein**	1.38 ± 0.109	1.52 ± 0.179	1.46 ± 0.178	1.56 ± 0.084	1.59 ± 0.164	1.70 ± 0.080	1.64 ± 0.111	1.64 ± 0.113
** Solids non-fat**	3.78 ± 0.272	4.16 ± 0.503	3.98 ± 0.492	4.20 ± 0.223	4.25 ± 0.402	4.55 ± 0.205	4.33 ± 0.265	4.24 ± 0.285
** Total solids**	5.26 ± 0.382	5.71 ± 0.597	5.52 ± 0.778	6.06 ± 0.354	6.12 ± 0.595	6.51 ± 0.316	6.22 ± 0.394	6.17 ± 0.448
**Milk solids, %**								
** Fat**	3.55 ± 0.129	3.56 ± 0.190	3.83 ± 0.083	4.00 ± 0.120	4.01 ± 0.120	3.93 ± 0.074	3.99 ± 0.132	4.27 ± 0.205
** Protein**	3.29 ± 0.064	3.29 ± 0.073	3.32 ± 0.054	3.36 ± 0.061	3.40 ± 0.085	3.41 ± 0.032	3.48 ± 0.089	3.59 ± 0.070
** Solids non-fat**	9.01 ± 0.067	9.00 ± 0.102	9.05 ± 0.053	9.04 ± 0.086	9.11 ± 0.150	9.12 ± 0.081	9.17 ± 0.145	9.31 ± 0.117
** Total solids**	12.6 ± 0.19	12.4 ± 0.23	12.5 ± 0.59	13.0 ± 0.18	13.1 ± 0.26	13.1 ± 0.15	13.2 ± 0.24	13.6 ± 0.28
**SCC, × 1000 cells/mL^c^**	275.4 ± 11.99	221.3 ± 12.62	212.3 ± 10.85	104.5 ± 13.06	100.0 ± 11.01	91.8 ± 11.01	104.7 ± 12.96	119.4 ± 11.67
**4% FCM, kg/day^d^**	35.8 ± 6.00	39.0 ± 7.89	38.0 ± 6.87	42.1 ± 7.23	42.6 ± 6.89	44.6 ± 7.00	38.8 ± 6.91	43.2 ± 7.03
**ECM, kg/day^e^**	39.8 ± 6.55	43.5 ± 7.97	42.2 ± 7.03	46.4 ± 7.41	47.1 ± 7.08	49.6 ± 7.17	44.4 ± 7.12	47.9 ± 7.31
**Adverse health events, %**	—	—	41.7 ± 11.3	31.8 ± 7.30	21.6 ± 3.53	29.2 ± 7.18	26.3 ± 8.79	19.4 ± 4.88
**Mortality rate, %**	—	—	2.22 ± 1.056	1.41 ± 0.729	0.89 ± 0.775	0.50 ± 0.436	0.83 ± 0.601	0.59 ± 0.465
**Conception rate, %**	—	—	40.2 ± 6.53	42.4 ± 7.42	40.3 ± 9.55	45.1 ± 5.54	38.4 ± 11.3	38.7 ± 12.40
**Abortion rate, %**	—	—	9.51 ± 1.89	9.40 ± 2.77	9.02 ± 2.42	11.2 ± 1.20	9.78 ± 4.44	7.29 ± 3.40

a Data from January to September 2023.

b Data from October, November, and December 2023.

c Data were log-transformed for further statistical analysis.

d 4% FCM calculated according to Gaines and Davidson (1923) as FCM = [(0.4 × milk yield) + (15 × fat yield)].

e ECM, standardized for 3.5% milk fat and 3.2% milk protein, was calculated according to Tyrell and Reid (1965) as ECM = [(12.82 × fat yield) + (7.13 × protein yield) + (0.323 × milk yield)].

### Overall productive performance

For all the variables regarding milk production and composition, the treatment × season interaction was not significant (*P* ≥ 0.08) and, therefore, only the main effects will be presented. Treatment group and season impacted milk yield and milk yield variation (*P* ≤ 0.02; Table 3). The supplementation of *B. licheniformis* 809 and *B. subtilis* 810 was associated with an increased milk yield (*P* = 0.02) in the order of 1.0 kg/cow per day (or 2.9%), while the milk yield variation was associated with a reduction of 0.29 kg/day (*P* < 0.0001) following the *Bacillus*-based DFM supplementation for 24 mo at the farm. Additionally, the DFM supplementation was associated with an increased 4% FCM and ECM yields (*P* ≤ 0.01) of 3.6 (+ 9.1%) and 4.1 (+ 9.4%) kg/day, respectively. For milk composition, supplementing lactating dairy cows in POST was also associated with a greater (*P* < 0.001) milk fat (+ 210 g or 12.4%), protein (+ 150 g or 10.0%), SNF (+ 250 g or 6.1%), and total solids yield (+ 510 g or 27.5), while SCC was not affected (*P* = 0.26; Table 3). For solids content (%), milk fat tended to be greater (*P* = 0.07), while milk protein (+ 0.12%-units), SNF (+ 0.12%-units), and total solids (+ 0.24%-units) were greater in POST vs. PRE (*P* ≤ 0.04).

**Table 3:** Milk yield, milk yield variation, and milk composition (yield and content) of lactating dairy cows before (*PRE*; 57 months) or after (*POST*; 24 months) the addition of a *Bacillus*-based direct-fed microbial (*DFM*) in the TMR over different seasons.^a^^,^^b^

Item	Group		SEM	Season				SEM	*P =^c^*		
	PRE	POST		Autumn	Winter	Spring	Summer		G	S	G × S
**Milk yield, kg/day**	35.0	36.0	1.99	34.8^x^	37.6^y^	37.1^y^	32.5^z^	2.01	0.02	<0.0001	0.63
**Milk yield variation, kg/day**	0.68	0.39	0.051	0.49^x^	0.46^x^	0.48^x^	0.72^y^	1.10	<0.0001	<0.0001	0.93
**Milk solids, kg/day**											
** Fat**	1.70	1.91	0.026	1.79^x^	1.97^y^	1.80^x^	1.66^x^	0.037	<0.0001	<0.0001	0.33
** Protein**	1.50	1.65	0.019	1.54^x^	1.70^y^	1.60^x^	1.44^z^	0.026	<0.0001	<0.0001	0.30
** Solids non-fat**	4.07	4.32	0.048	4.07^xz^	4.59^y^	4.30^xy^	3.87^z^	0.066	<0.001	<0.0001	0.21
** Total solids**	5.72	6.23	0.073	5.83^xz^	6.49^y^	6.11^xy^	5.48^z^	0.099	<0.0001	<0.0001	0.42
**Milk solids, %**											
** Fat**	3.97	4.07	0.027	4.07^xy^	4.11^y^	3.95^xz^	3.89^z^	0.034	0.07	<0.001	0.08
** Protein**	3.37	3.49	0.014	3.46^y^	3.46^y^	3.43^xy^	3.37^x^	0.019	<0.0001	<0.001	0.23
** Solids non-fat**	9.07	9.19	0.021	9.17^y^	9.20^y^	9.13^xy^	9.04^x^	0.027	<0.01	<0.001	0.29
** Total solids**	12.95	13.19	0.072	13.15^y^	13.27^y^	13.11^xy^	12.76^x^	0.091	0.04	<0.01	0.84
**SCC, × 1000 cells/mL^d^**	128.8	114.8	10.28	120.2^xy^	107.2^x^	109.6^xy^	147.9^y^	10.40	0.26	0.03	0.75
**4% FCM, kg/day^e^**	39.5	43.1	2.12	40.8^x^	44.6^y^	41.8^x^	37.9^z^	2.42	0.01	<0.0001	0.59
**ECM, kg/day^f^**	43.8	47.9	2.27	48.2	49.5	46.5	42.0	2.46	<0.01	<0.0001	0.61

a The *Bacillus*-based direct fed microbial (Bovacillus; Novonesis, Lyngby, Denmark) was offered at 3 g/cow per day.

b Different letters denote differences at *P* ≤ 0.05 level for the “season” effect.

c *G* = group effect (PRE and POST); *S* = season effect; *G × S* = group × season.

d Data were log-transformed for further statistical analysis.

e 4% FCM calculated according to Gaines and Davidson (1923) as FCM = [(0.4 × milk yield) + (15 × fat yield)].

f ECM, standardized for 3.5% milk fat and 3.2% milk protein, was calculated according to Tyrell and Reid (1965) as ECM = [(12.82 × fat yield) + (7.13 × protein yield) + (0.323 × milk yield)].

Cows in the winter and spring produced more milk (*P* < 0.01) than in the fall and summer, but also greater milk yield for cows in the autumn vs. summer (*P* < 0.01). Moreover, season of the year significantly impacted both yield (*P* < 0.0001) and content (*P* < 0.001; Table 3), as cows in winter produced more solids than in all other seasons (*P* ≤ 0.05), while cows in the summer had the lowest milk solids yield and content (*P* ≤ 0.05).

### Health status

For the health parameters, no treatment × season interaction was noted (*P* ≥ 0.63) for either the occurrence of adverse health events or the mortality rate (Table 4). However, main effects of treatment were observed for both variables (*P* < 0.001), while season impacted only the occurrence of adverse health events (*P* < 0.01). A reduction in the occurrence of adverse heath events (32.8 vs. 23.7% for PRE and POST, respectively) and culling rate (1.6 vs. 0.7% for PRE and POST, respectively) was associated with the DFM feeding. Among the individual adverse health outcomes, *Bacillus* spp. supplementation in the POST period was associated with reduction in mastitis (−1.9%-units; *P* = 0.02), ketosis (−1.5%-units; *P* < 0.01), metritis (−3.7%-units; *P* < 0.0001), displaced abomasum (−0.4%-units; *P* < 0.01), and pneumonia (−0.7%-units; *P* < 0.01), while the same association effect tended to be observed for retained placenta (−0.8%-units; *P* = 0.10). Nonetheless, opposite associative results tended to be observed for the occurrence of lameness (+ 1.2%-units; *P* = 0.07; Table 4) in POST vs. PRE groups.

**Table 4:** Occurrence of adverse health events, culling rate, conception rate, and abortion rate of lactating dairy cows before (*PRE*; 57 months) or after (*POST*; 24 months) the addition of a *Bacillus*-based direct-fed microbial (*DFM*) in the TMR over different seasons.^a^^,^^b^

Item	Group		SEM	Season				SEM	*P =^c^*		
	PRE	POST		Autumn	Winter	Spring	Summer		G	S	G × S
**Occurrence of adverse health events, %**	32.8	23.7	1.74	22.6^x^	24.6^x^	31.5^xy^	34.2^y^	2.51	<0.001	<0.01	0.84
** Metritis**	10.8	6.1	0.71	6.9^x^	6.5^x^	9.5^xy^	11.1^y^	1.03	<0.0001	<0.01	0.98
** Mastitis**	7.5	5.6	0.55	5.3^x^	4.8^x^	7.8^xy^	8.4^y^	0.80	0.02	<0.01	0.73
** Retained placenta**	5.7	4.5	0.51	3.1^x^	4.3^x^	7.7^y^	5.3^xy^	0.07	0.10	<0.001	0.14
** Lameness**	4.2	5.4	0.48	4.4	4.5	4.3	6.1	0.65	0.07	0.19	0.58
** Ketosis**	1.8	0.3	0.32	0.9	0.9	0.7	1.7	0.45	<0.01	0.42	0.79
** Hypocalcemia**	1.4	1.4	0.19	1.4^x^	2.6^y^	0.9^x^	0.7^x^	0.03	0.86	<0.0001	0.15
** Pneumonia**	1.0	0.3	0.13	0.6	0.8	0.7	0.5	0.20	<0.01	0.79	0.72
** Displaced abomasum**	0.4	0.0	0.09	0.1	0.2	0.0	0.3	0.12	<0.01	0.54	0.71
**Mortality rate, %**	1.6	0.7	0.16	1.12	1.18	1.00	1.26	0.228	<0.001	0.89	0.62
**Conception rate, %**	41.0	39.7	0.92	44.7^y^	46.8^y^	42.3^y^	27.4^x^	1.33	0.31	<0.0001	0.03
**Abortion rate, %**	9.5	8.9	0.47	8.5^x^	7.4^x^	12.3^y^	8.5^x^	0.68	0.42	<0.0001	<0.01

a The *Bacillus*-based direct fed microbial (Bovacillus; Novonesis, Lyngby, Denmark) was offered at 3 g/cow per day.

b Different letters denote differences at *P* ≤ 0.05 level for the “season” effect.

c *G* = group effect (PRE and POST); *S* = season effect; *G × S* = group × season.

Cows in the summer presented a greater occurrence of adverse health events vs. cohorts in the autumn and winter (*P* ≤ 0.03). The occurrence of metritis and mastitis were also greater for cows in the summer (*P* ≤ 0.05), while retained placenta was greater in the spring (*P* ≤ 0.04) and hypocalcemia was greater in the winter (*P* ≤ 0.04; Table 4).

### Reproductive performance

Treatment group × season interactions were observed for both conception and abortion rates (*P* ≤ 0.03; Fig. 1); however, the interactions were noted mainly because of the season effect, given that within the same season, no treatment differences were observed (*P* ≥ 0.14). Conception rates were greater (*P* ≤ 0.05) in the autumn, winter, and spring vs. summer, while abortion rates were greater in the spring vs. all other seasons (*P* ≤ 0.03). No treatment group effects were observed either on conception (*P* = 0.31) or abortion rate (*P* = 0.42; Table 4).

**Figure 1:**
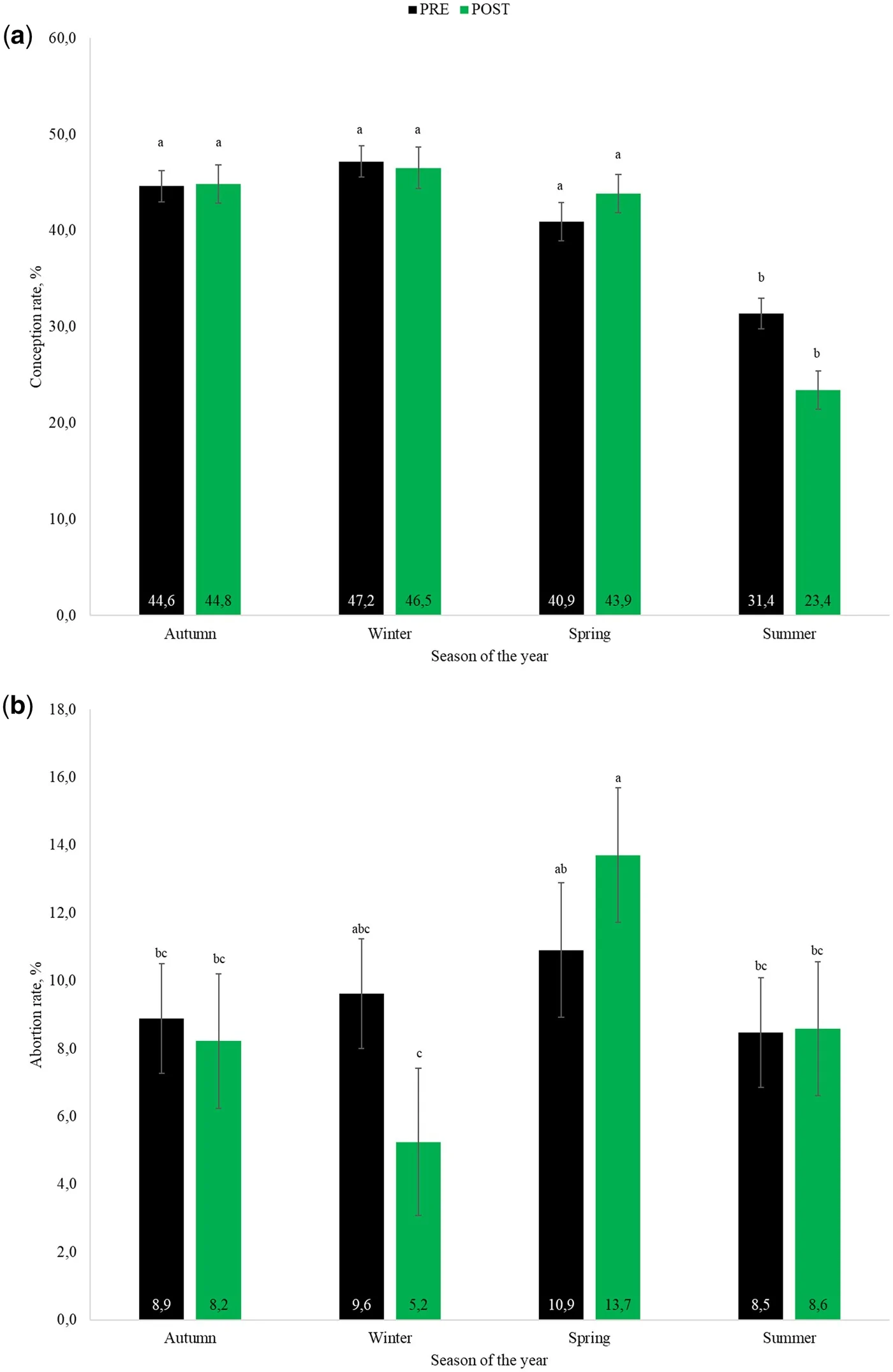
Treatment × season interactions observed in conception (*1-a*) and abortion (*1-b*) rates observed in the current retrospective before-and-after analysis of supplementing *Bacillus licheniformis* 809 and *B. subtilis* 810 for 24 months (*POST*) when compared with the previous 57 months of the supplementation (*PRE*). Treatment × season interactions were reported for both variables (*P* ≤ 0.03). ^a–c^Different letters denote differences at *P* ≤ 0.05.

### Additional performance, health, and reproductive analysis (48-month period)

Due to the changes in the dietary management reported in 2023 until the end of the data analysis, an additional analysis was performed evaluating 24 months PRE with the 24 months POST the DFM feeding. Milk yield (34.8 vs. 36.0 kg for PRE and POST, respectively; SEM = 2.11; *P* = 0.02), milk variation (0.58 vs. 0.39 kg for PRE and POST, respectively; SEM = 0.106; *P* < 0.01), 4% FCM (41.7 vs. 43.1 kg for PRE and POST, respectively; SEM = 2.88; *P* = 0.04), ECM (46.1 vs. 47.9 kg for PRE and POST, respectively; SEM = 3.11; *P* = 0.05), milk fat yield (1.85 vs. 1.91 kg for PRE and POST, respectively; SEM = 0.018; *P* = 0.04), milk protein content (3.39 vs. 3.49% for PRE and POST, respectively; SEM = 0.016; *P* < 0.001) and yield (1.57 vs. 1.65 kg for PRE and POST, respectively; SEM = 0.014; *P* < 0.01), solids non-fat content (9.08 vs. 9.19% for PRE and POST, respectively; SEM = 0.024; *P* < 0.01) and yield (4.23 vs. 4.32 kg for PRE and POST, respectively; SEM = 0.037; *P* < 0.01), total solids content (13.1 vs. 13.2% for PRE and POST, respectively; SEM = 0.04; *P* = 0.02) and yield (6.08 vs. 6.23 kg for PRE and POST, respectively; SEM = 0.052; *P* < 0.01) were still associated with the supplementation of *B. licheniformis* 809 and *B. subtilis* 810. On the other hand, milk fat content (*P* = 0.16) and milk SCC (*P* = 0.63) did not differ from PRE and POST over this 48-month retrospective analysis.

For health events, DFM feeding in the POST period remained positively-associated with a reduction in the occurrence of adverse health events (29.0 vs. 23.7%; SEM = 1.48; *P* = 0.02) and the mortality rate (1.2 vs. 0.7%; SEM = 0.13; *P* = 0.01). For the specific adverse health events, mastitis tended to be associated with a reduction due to the DFM supplementation in the POST period (7.2 vs. 5.6%; SEM = 0.61; *P* = 0.06), while metritis (9.2 vs. 6.1%; SEM = 0.62; *P* < 0.01), ketosis (1.0 vs. 0.3%, SEM = 0.16; *P* < 0.01), pneumonia (1.0 vs. 0.3%; SEM = 0.14; *P* < 0.01), and displaced abomasum (0.2 vs. 0.0%; SEM = 0.04; *P* = 0.03) were reduced and associated with DFM feeding. Lastly, lameness, hypocalcemia, and retained placenta occurrence did not differ between PRE and POST (*P* ≥ 0.13). Similarly, no differences between PRE and POST were observed on either conception (*P* = 0.21) or abortion (*P* = 0.61) rates.

## Discussion

The assessment of the efficacy of different technologies under the farm conditions may not be feasible or may be more difficult, primarily due to the daily challenges often observed, including those associated with the environment, diet, health, lactation stage, genetics, and other potential managements not anticipated. Hence, the assessment of these potential benefits, if any, should be validated over an extended period of time so that the effects, either positive or negative, of any significant variation between years can be diluted over time. Based on that, we conducted a long-term before-and-after retrospective evaluation of milk production, milk composition, health, and reproductive performance of a commercial dairy cow farm located in Argentina prior to (57 months) and after (24 months) the inclusion of a *Bacillus*-based DFM in the TMR for the entire herd. A secondary analysis also considered 24 months prior to feeding, as the farm itself was improving its own productive and health parameters, as previously presented. It is noteworthy mentioning that the current results are not to be overinterpreted by the reader, as a negative control was not concurrently assessed with the *Bacillus*-based DFM supplementation period; therefore, causality cannot be implied here, while the current results should be seen and evaluated as (i) supportive results to the recent scientific literature of this probiotic mixture in early-, mid-, and late-lactating dairy cows (Oyebade et al. 2023, 2024; Cappellozza et al. 2024; Terré et al. 2024; Arthur et al. 2025; Juckem et al. 2025; Bach et al. 2026; Nicola et al. 2026) and (ii) the potential association of the probiotic supplementation with the productive and reproductive performance, as well as health status of a commercial dairy farm over a long-period of time. Direct-fed microbials are known as microbial solutions that benefit the health of the host when offered in the recommended dosage (FAO/WHO 2001). The fact that health is explicitly mentioned in the definition of a DFM, but not necessarily performance, implies that health drives the performance results of the herd. In agreement with this statement that health will impact short-, medium-, and long-term performance of dairy cows, Carvalho et al. (2019) demonstrated that dairy cows without clinical diseases until day 21 post-partum had a greater daily milk yield during the entire lactation, peak milk yield, solids yield, and reproductive function when compared to cows showing ≥1 adverse health events.

In the current retrospective study, feeding the *Bacillus*-based DFM for 24 months was associated with a reduced mean occurrence of adverse health events (9.1%-units) and mortality rates when compared with the 57- or 24-months before DFM feeding. More specifically, cases of metritis, mastitis, ketosis, retained placenta, pneumonia, and displaced abomasum were significantly associated with a reduced occurrence when the DFM was fed for 24 months vs. the previous 57 months. When the analysis considered 24 moths PRE, occurrence of metritis, ketosis, pneumonia, and displaced were associated with the DFM feeding POST. Nonetheless, opposite results were observed for occurrence of lameness for 57 months, but not for the 24 months analysis. The current results support previous scientific and retrospective studies in dairy cows and calves. However, the improvement in the disease outcomes reported herein could also have been affected by any change in farm management and disease surveillance, highlighting the lack of causality from these findings. Using a similar retrospective design as herein, Fensterseifer et al. (2022) demonstrated that *B. subtilis* and *B. pumilus* feeding (10 g/cow per day; 4.9 × 10^9^ colony forming units/cow per day) for 120 days was associated with a reduced occurrence of metritis and mastitis when compared with the pre-Bacilli feeding period. Recently, the supplementation of *B. licheniformis* 809 and *B. subtilis* 810 to multiparous Holstein dairy cows from 30 days pre-partum until 60 days post-partum significantly reduced the SCC and the proportion of cows with SCC > 200,000 cells/mL, while increasing serum immunoglobulin A (*IgA*) concentration at calving (Nicola et al. 2026). One might speculate the effects on mastitis occurrence when compared with the lack of significance on milk SCC; however, it is important to mention that the milk SCC was evaluated as a milk bulk tank sample, while mastitis occurrence was evaluated per animal at the moment of milking. In pre-weaning calves, the occurrence of gastrointestinal and respiratory adverse health events was reduced when *B. licheniformis* 809 and *B. subtilis* 810 was fed via whole milk or milk replacer (Magalhães et al. 2024; Terré et al. 2025). The results on lameness were unexpected and their potential association with the *Bacillus*-based probiotic feeding, if any, is unknown. However, the occurrence reported herein is at the lower end of occurrence, as reported by Thomsen et al. (2023). Nonetheless, the quality and management of the lying surface might significantly lead to higher or lower occurrence of lameness, as explained and summarized by Wessels et al. (2025), while the personnel performing the locomotion score could also have impacted these results, as previously mentioned.

In beef heifers receiving a LPS challenge, *Bacillus* spp. supplementation modulated the innate immune response by reducing rectal temperature, plasma cortisol, and haptoglobin at specific timepoints after LPS administration. Moreover, mean plasma concentrations of tumor necrosis factor-alpha and enzymes associated with liver function were also reduced in heifers fed the DFM (Mackey et al. 2025). The modes of action leading to these results might be associated with the modulation of the immune response, such that it might be weakened after the occurrence of a challenge, so that the feed intake resumption is faster and the expenditure of nutrients caused by the activation of the innate immune response is likely minimized following the feeding of a *Bacillus*-based DFM (Goetz et al. 2023, 2024; Mackey et al. 2025). Moreover, the support of a technology like the *Bacillus*-based DFM used herein also involves the support to the integrity of the intestinal epithelial cells, by alleviating the severity and occurrence of leaky gut (Boll et al. 2024), formation of biofilm (Segura et al. 2020), production of secreted and membrane-bound mucins (Santano et al. 2020), inhibition of potentially harmful bacteria (Capern et al. 2025, 2026), and maintenance of the oxidative balance in the gastrointestinal tract lumen and blood (Cordeiro et al. 2024; Mackey et al. 2025) of ruminants.

In the current study, beneficial results on milk production and milk composition were associated with the DFM supplementation for 24 months, regardless if the PRE period comprised 57 or 24 months. The improvements in milk yield ranged from 1.0 to 1.2 kg/day, 4% FCM ranged from 1.4 to 3.6 kg/day, and ECM ranged from 1.8 to 4.1 kg/day when the 24 and 57 months of assessment were respectively taken, while positive effects were also observed on milk composition. Supplementing lactating dairy cows with *B. licheniformis* 809 and *B. subtilis* 810 in randomized controlled studies improved milk yield (1.2 to 1.3 kg; Cappellozza et al. 2024; Bach et al. 2026), feed efficiency (40 to 190 g milk/kg feed; Cappellozza et al. 2024; Terré et al. 2024; Bach et al. 2026), milk fat (40 g; Bach et al. 2026), protein (30 to 50 g; Cappellozza et al. 2024; Bach et al. 2026), and lactose (60 to 80 g; Cappellozza et al. 2024; Bach et al. 2026). Fat-corrected milk yield, milk fat %, and FCM feed efficiency have also increased when the same *Bacillus* spp. combination was added to a DFM containing *Lactobacillus animalis* and *Propionibacterium freudenreichii* (Oyebade et al. 2023). In transition dairy cows, milk yield and FCM increased by 0.7 and 0.9 kg/cow per day, respectively, when *B. licheniformis* 809 and *B. subtilis* 810 was fed from 30 days pre-partum until 60 days post-partum (Nicola et al. 2026), while feed efficiency and milk fat % also increased in mid- to late-lactating dairy cows (Arthur et al. 2025). Besides the health-supportive effects, *Bacillus* spp. secretes digestive enzymes (Schallmey et al. 2004; Luise et al. 2022), explaining the improved in vitro (Pan et al. 2022; Cappellozza et al. 2023), *in situ* (Arthur et al. 2025; Limede et al. 2025, 2026), and in vivo (Terré et al. 2025; Nicola et al. 2026; De Souza et al. 2026) nutrient digestibility. Previous authors have demonstrated the importance of improving rumen and total-tract nutrient digestibility in lactating dairy cows (Oba and Allen 1999; Ferraretto et al. 2013), partially explaining the improved milk production and milk composition observed in the current farm study. It is noteworthy mentioning that the improvements in milk production and composition were observed without negative effects on the reproductive performance of the dairy cow herd. Altogether the results from the current retrospective study follow the same positive association following the feeding of the DFM, although no true causal relationship can be drawn from the current dataset.

Although the current study was performed in a single farm in Argentina with Holstein cows housed in free-stall barns, the productive and health responses follow the scientific, randomized, and controlled publications using Holstein cows in compost bedding (Nicola et al. 2026) or free-stall housed cows offered different types of diets (Oyebade et al. 2023; Cappellozza et al. 2024; Terré et al. 2024; Bach et al. 2026), with different breeds (Cappellozza et al. 2024), and in different environmental conditions as used herein (tropical and temperate climate; Oyebade et al. 2023; Cappellozza et al. 2024; Terré et al. 2024; Arthur et al. 2025; Bach et al. 2026).

As expected, HS was present in this retrospective observation and, therefore, season effects were observed for most of the productive, health, and reproductive variables analyzed herein. The milk production, composition, health, and reproductive function were impaired in the summer when compared with the other seasons, likely due to the occurrence of heat stress. Previous authors demonstrated that heat stress negatively impacts the metabolism (Rhoads et al. 2010; Gao et al. 2017), reproduction (Pereira et al. 2021), health (Leiva et al. 2017), and productive (West 2003) performance of the dairy cow herd.

In summary, this retrospective before-and-after retrospective study indicated that milk yield, composition, and health were positively associated with the supplementation of the herd with *B. licheniformis* 809 and *B. subtilis* 810, while its reproductive performance was maintained. A couple of limitations herein include (i) the fact that this was a retrospective study without a contemporary supplemented and non-supplemented groups, no definite causality can be drawn from the current results and (ii) the external confounding factors, such as the environment, genetics, and changes in the nutritional profile of the diets, might have impacted our results. Nonetheless, one strength of our dataset is that it represents a practical adoption of a nutritional technology in a commercial dairy farm over a long-term period. Additional studies are warranted to further assess the application of a DFM in the farm, enrolling additional farms under different management conditions and breeds.

## Funding

None declared.

## Notes

All animals were cared for in accordance with FASS (2020).

## List of abbreviations

DFM: direct-fed microbial
HS: heat stress
POST: period which the Bacillus-based probiotic diet was fed
PRE: period which the unsupplemented diet was fed
TAI: timed-artificial insemination
THI: temperature-humidity index
TMR: total mixed ration

## References

[txag144-B1] Arthur B. A. V. et al 2025. *Bacillus* spp. supplementation promotes feed efficiency in mid- to late-lactation dairy cows and impacts rumen fermentation traits of rumen-fistulated females offered a corn silage-based TMR diet. JDS Commun. 6:490–496. 10.3168/jdsc.2024-0686 PMC12848273 41613208

[txag144-B2] Bach A. et al 2026. Effects of supplementing a *Bacillus*-based direct-fed microbial on ex vivo fermentation traits and on performance of lactating Holstein dairy cows. J. Dairy Sci. 109:1187–1201. 10.3168/jds.2025-27134 41397615

[txag144-B94731660] Bernardeau M., , Lehtinen M. J., Forssten S. D., Nurminen P. 2017. Importance of the gastrointestinal life cycle of Bacillus for probiotic functionality. J. Food Sci. Technol. 54:2570–2584. 10.1007/s13197-017-2688-3 PMC5502041 28740315

[txag144-B3] Boll E. J. , Copani G., Cappellozza B. I. 2024. *Bacillus paralicheniformis* 809 and *Bacillus subtilis* 810 support in vitro intestinal integrity under hydrogen peroxide and deoxynivalenol challenges. Transl. Anim. Sci. 8:txae061. 10.1093/tas/txae061 PMC11056882 38685987

[txag144-B4] Capern L. et al 2026. Co-incubation of Bacillus spp. alleviated the growth and gas production of potentially harmful bacteria in different in vitro methodologies. JDS Commun. 7:601–607. 10.3168/jdsc.2025-0896 PMC13542424 42698911

[txag144-B5] Capern L. C. , Gresse R., Queiroz O. C. M., Cappellozza B. I. 2025. *Bacillus licheniformis* 809 and *Bacillus subtilis* 810 promote in vitro supportive effects against potentially harmful microorganisms under a feed matrix-based assay. J. Anim. Sci. 103. 10.1093/jas/skaf186 PMC12288027 40443021

[txag144-B6] Cappellozza, B. I . et al 2025. Ruminant nutrition symposium: novel microbial solutions to optimize production efficiency in beef and dairy systems. J. Anim. Sci. 103:skaf165. 10.1093/jas/skaf165 PMC12202103 40372016

[txag144-B7] Cappellozza B. I. et al 2023. Evaluation of a *Bacillus*-based direct-fed microbial probiotic on in vitro rumen gas production and nutrient digestibility of different feedstuffs and total mixed rations. Transl. Anim. Sci. 7:txad044. 10.1093/tas/txad044 PMC10199785 37216187

[txag144-B8] Cappellozza B. I. et al 2024. Effects of supplementing a *Bacillus*-based direct-fed microbial on performance, nutrient digestibility, rumen fermentation characteristics, and metabolic responses of lactating dairy cows. JDS Commun. 5:107–112. 10.3168/jdsc.2023-0439 PMC10928428 38482120

[txag144-B9] Carvalho M. R. et al 2019. Long-term effects of postpartum clinical disease on milk production, reproduction, and culling of dairy cows. J. Dairy Sci. 102:11701–11717. 10.3168/jds.2019-17025 31548073

[txag144-B10] Cordeiro M. W. S. , Cappellozza B. I., de Melo N. N., Bernardes T. F. 2024. Effects of a *Bacillus*-based direct-fed microbial on performance, blood parameters, fecal characteristics, rumen morphometrics, and intestinal gene expression in finishing beef bulls. J. Anim. Sci. 102:skae259. 10.1093/jas/skae259 PMC11439149 39248595

[txag144-B51] De Souza I. S. et al 2026. Effects of feeding a *Bacillus*-based probiotic on nutrient utilization and microbiota profile in the rumen and reproductive tract of beef heifers receiving a forage-based diet. J. Anim. Sci. 104:skaf411. 10.1093/jas/skaf411 PMC12863943 41317362

[txag144-B11] El Jeni R. et al 2024. Invited review: “Probiotic” approaches to improving dairy production: reassessing “magic foo-foo dust”. J. Dairy Sci. 107:1832–1856. 10.3168/jds.2023-23831 37949397

[txag144-B12] FAO/WHO. 2001. Probiotics in food: health and nutritional properties of probiotics in food including powder milk with live lactic acid bacteria. World Health Organization. https://www.fao.org/3/a0512e/a0512e.pdf

[txag144-B13] FASS. 2020. Guide for care and use of agricultural animals in research and teaching. 4th ed. American Dairy Science Association, American Society of Animal Science, and Poultry Science Association, Champaign, IL.

[txag144-B14] Fernandes L. , Guimaraes I., Noyes N. R., Caixeta L. S., Machado V. S. 2021. Effect of subclinical mastitis detected in the first month of lactation on somatic cell count linear scores, milk yield, fertility, and culling of dairy cows in certified organic herds. J. Dairy Sci. 104:2140–2150. 10.3168/jds.2020-19153 33309348

[txag144-B15] Fensterseifer S. R. , Arias R. P., Peter C. M., Lange A., Galbraith E. 2022. Effects of *bacillus*-based direct-fed microbial on health and production of high-performing lactating dairy cows. J. Dairy Sci. 105:44. (Abstr.)

[txag144-B16] Ferraretto L. F. , Crump P. M., Shaver R. D. 2013. Effect of cereal grain type and corn grain harvesting and processing methods on intake, digestion, and milk production by dairy cows through a meta-analysis. J. Dairy Sci. 96:533–550. 10.3168/jds.2012-5932 23164230

[txag144-B17] Gaines W. L. , Davidson F. A. 1923. Relation between percentage of fat content and yield of milk: correction of milk yield for fat content. University of Illinois Agricultural Experiment Station Bulletin, Urbana, IL. https://hdl.handle.net/2142/3304

[txag144-B18] Gao S. T. et al 2017. The effects of heat stress on protein metabolism in lactating Holstein cows. J. Dairy Sci. 100:5040–5049. 10.3168/jds.2016-11913 28390717

[txag144-B19] Garzon A. et al 2022. Defining clinical diagnosis and treatment of puerperal metritis in dairy cows: a scoping review. J. Dairy Sci. 105:3440–3452. 10.3168/jds.2021-21203 35151476

[txag144-B20] Goetz B. M. et al 2023. Effects of *Bacillus subtilis* PB6 supplementation on production, metabolism, inflammatory biomarkers, and gastrointestinal tract permeability in transition dairy cows. J. Dairy Sci. 106:9793–9806. 10.3168/jds.2023-23562 37641308

[txag144-B21] Goetz B. M. et al 2024. Effects of a multistrain *Bacillus*-based direct-fed microbial on gastrointestinal permeability and biomarkers of inflammation during and following feed restriction in mid-lactation Holstein cows. J. Dairy Sci. 107:6192–6210. 10.3168/jds.2023-24352 38395402

[txag144-B22] Haimerl P. , Heuwieser W. 2014. Invited review: antibiotic treatment of metritis in dairy cows: a systematic approach. J. Dairy Sci. 97:6649–6661. 10.3168/jds.2014-8462 25218751

[txag144-B23] Juckem K. A. , Pupo M. R., Diepersloot E. C., Ferraretto L. F. 2025. Effect of dietary starch concentration and direct-fed microbial supplementation on lactation performance, total-tract nutrient digestibility, and enteric methane emissions by dairy cows. J. Dairy Sci. 108:12257–12274. 10.3168/jds.2025-26694 40912559

[txag144-B24] Le Blanc S. J. , Leslie K. E., Duffield T. F. 2005. Metabolic predictors of displaced abomasum in dairy cattle. J. Dairy Sci. 88:159–170. 10.3168/jds.S0022-0302(05)72674-6 15591379

[txag144-B25] Leiva T. et al 2017. Supplementing an immunomodulatory feed ingredient to modulate thermoregulation, physiologic, and production responses in lactating dairy cows under heat stress conditions. J. Dairy Sci. 100:4829–4838. 10.3168/jds.2016-12258 28434746

[txag144-B26] Limede A. C. et al 2025. Effects of a *Bacillus*-based direct-fed microbial on digestibility, ruminal in situ nutrient disappearance, microbiome, and fermentation parameters in forage-fed beef cattle. J. Anim. Sci. 103:skaf093. 10.2527/jas.2024-9302 PMC12065412 40139973

[txag144-B27] Limede A. C. et al 2026. Effects of *Bacillus licheniformis* 809 and *Bacillus subtilis* 810 supplementation on in situ degradation of a high-concentrate byproduct-based diet in forage-fed beef cows. Transl. Anim. Sci. 10:txag055. 10.1093/tas/txag055 PMC13198368 42180804

[txag144-B28] Luise D. et al 2022. Bacillus spp. probiotic strains as a potential tool for limiting the use of antibiotics and improving the growth and health of pigs and chickens. Front. Microbiol. 13:801827. 10.3389/fmicb.2022.801827 PMC8859173 35197953

[txag144-B30] Mackey S. J. et al 2025. Feeding a *Bacillus*-based probiotic to beef heifers during stress challenges: effects on feed intake, immune, and metabolic responses. J. Anim. Sci. 103:skaf416. 10.1093/jas/skaf416 PMC12715315 41335446

[txag144-B31] Mader T. L. , Davis M. S., Brown-Brandl T. 2006. Environmental factors influencing heat stress in feedlot cattle. J. Anim. Sci. 84:712–719. 10.2527/2006.843712x 16478964

[txag144-B32] Magalhães J. et al 2024. Effects of supplementing direct-fed microbials on health and growth of preweaning Gyr × Holstein dairy calves. J. Dairy Sci. 107:6117–6130. 10.3168/jds.2023-24434 38608942

[txag144-B33] Nehme Marinho M. et al 2024. Dietary supplementation of rumen native microbes improves lactation performance and feed efficiency in dairy cows. J. Dairy Sci. 107:7918–7931. 10.3168/jds.2024-24795 38876222

[txag144-B34] McArt J. A. A. , Nydam D. V., Oetzel G. R. 2012. Epidemiology of subclinical ketosis in early lactation dairy cattle. J. Dairy Sci. 95:5056–5066. 10.3168/jds.2012-5443 22916909

[txag144-B35] McCarthy M.-C. , O’Grady L., McAloon C. G., Mee J. F. 2021. The effect of contract-rearing on the health status of replacement dairy heifers. Animals (Basel). 11:3447. 10.3390/ani11123447 PMC8698005 34944224

[txag144-B36] NASEM. 2021. Nutrient requirements of dairy cattle. 8th rev. ed. Natl. Acad. Press, Washington, DC. 10.17226/25806

[txag144-B37] Nicola M. S. et al 2026. Effects of supplementation with *Bacillus licheniformis* 809 and *Bacillus subtilis* 810 on colostrum quality, performance, digestibility, and metabolism of dairy cows in the transition period. J. Dairy Sci. 109:2400–2411. 10.3168/jds.2025-27305 41570895

[txag144-B38] Oba M. , Allen M. S. 1999. Evaluation of the importance of the digestibility of neutral detergent fiber from forage: effects on dry matter intake and milk yield of dairy cows. J. Dairy Sci. 82:589–596. 10.3168/jds.S0022-0302(99)75271-9 10194678

[txag144-B39] Oetzel G. R. 2004. Monitoring and testing dairy herds for metabolic disease. Vet. Clin. North Am. Food Anim. Pract. 20:651–674. 10.1016/j.cvfa.2004.06.006 15471629

[txag144-B40] Oetzel G. R. , Miller B. E. 2012. Effect of oral calcium bolus supplementation on early-lactation health and milk yield in commercial dairy herds. J. Dairy Sci. 95:7051–7065. 10.3168/jds.2012-5510 23040027

[txag144-B41] Ouellet V. , Toledo I. M., Dado-Senn B., Dahl G. E., Laporta J. 2021. Critical Temperature-Humidity Index thresholds for dry cows in a subtropical climate. Front. Anim. Sci. 2:706636. 10.3389/fanim.2021.706636

[txag144-B42] Oyebade A. O. et al 2024. Effects of direct-fed microbial supplement on ruminal and plasma metabolome of early-lactation dairy cows: untargeted metabolomics approach. J. Dairy Sci. 107:2556–2571. 10.3168/jds.2023-23876 37939839

[txag144-B43] Oyebade A. O. et al 2023. Effects of direct-fed microbial supplementation on performance and immune parameters of lactating dairy cows. J. Dairy Sci. 106:8611–8626. 10.3168/jds.2022-22898 37641244

[txag144-B44] Pan L. , Harper K., Queiroz O., Copani G., Cappellozza B. I. 2022. Effects of a *Bacillus*-based direct-fed microbial on in vitro nutrient digestibility of forage and high-starch concentrate substrates. Transl. Anim. Sci. 6:txac067. 10.1093/tas/txac067 PMC9186312 35702175

[txag144-B45] Pereira M. H. C. Jr , et al 2021. Increasing the length of an estradiol with progesterone timed artificial insemination protocol with 2 controlled internal drug release devices improves pregnancy per artificial insemination in lactating dairy cows. J. Dairy Sci. 104:1073–1086. 10.3168/jds.2020-18872 33131810

[txag144-B46] Rhoads M. L. et al 2010. Effects of heat stress and nutrition on lactating Holstein cows: II. Aspects of hepatic growth hormone responsiveness. J. Dairy Sci. 93:170–179. 10.3168/jds.2009-2469 20059916

[txag144-B47] Santano N. B. , Boll E. J., Capern L. C. 2020. Comparative evaluation of the antimicrobial and mucus induction properties of selected Bacillus strains against enterotoxigenic *Escherichia coli*. Antibiotics (Basel). 9:849. 10.3390/antibiotics9120849 PMC7760508 33261022

[txag144-B48] Schallmey M. , Singh A., Ward O. P. 2004. Developments in the use of Bacillus species for industrial production. Can. J. Microbiol. 50:1–17. htpps://doi.org/10.1139/w03-076 10.1139/w03-076 15052317

[txag144-B49] Schlageter-Tello A. et al 2014. Manual and automatic locomotion scoring systems in dairy cows: a review. Prev. Vet. Med. 116:12–25. 10.1016/j.prevetmed.2014.06.006 25000863

[txag144-B50] Segura A. , Milora N., Queiroz O., Cantor M. D., Copani G. 2020. In vitro evaluation of *Bacillus licheniformis* and *Bacillus subtilis* enzyme activity, *Clostridium perfringens* type a inhibition, and biofilm formation. J. Dairy Sci. 103(Suppl. 1):89. (Abstr.)

[txag144-B52] Souza V. L. et al 2017. Lactation performance and diet digestibility of dairy cows in response to the supplementation of *Bacillus subtilis* spores. Livest. Sci. 200:35–39. 10.1016/j.livsci.2017.03.023

[txag144-B53] Terré M. et al 2024. Supplementing a *Bacillus*-based direct-fed microbial improves feed efficiency in lactating dairy cows. Transl. Anim. Sci. 8:txae110. 10.1093/tas/txae110 PMC11316034 39131203

[txag144-B54] Terré M. , Queiroz O. C. M., Joergensen J. N., Cappellozza B. I. 2025. *Bacillus* spp. supplementation promotes feed efficiency and alters metabolic responses of pre-weaning Angus × Holstein calves. J. Dairy Sci. 108:10888–10899. 10.3168/jds.2025-26866 40780653

[txag144-B55] Thomsen P. T. , Shearer J. K., Houe H. 2023. Prevalence of lameness in dairy cows: a literature review. Vet. J. 295:105975. 10.1016/j.tvjl.2023.105975 36990338

[txag144-B56] Tyrell H. F. , Reid J. T. 1965. Prediction of the energy value of cow’s milk. J. Dairy Sci. 48:1215–1223. 10.3168/jds.S0022-0302(65)88430-2 5843077

[txag144-B57] Valldecabres A. et al 2022. Effects of rumen-native microbial feed supplementation on milk yield, composition, and feed efficiency in lactating dairy cows. J. Anim. Sci. 100:skac275. 10.1093/jas/skac275 PMC9584157 36041454

[txag144-B58] Wessels W. et al 2025. A systematic review and meta-analysis of lameness scoring methods and the prevalence of lameness of dairy cows in Northwest Europe. J. Dairy Sci. 108:10037–10059. 10.3168/jds.2025-26585 40614806

[txag144-B59] West J. W. 2003. Effects of heat-stress on production in dairy cattle. J. Dairy Sci. 86:2131–2144. 10.3168/jds.S0022-0302(03)73803-X. 12836950

